# In primary total hip arthroplasty, the direct anterior approach leads to higher levels of creatine kinase and lower levels of C-reactive protein compared to the posterolateral approach: a propensity score matching analysis of short-term follow-up data

**DOI:** 10.1186/s13018-023-04084-x

**Published:** 2023-08-11

**Authors:** Luca De Berardinis, Marco Senarighi, Luca Farinelli, Fjorela Qordja, Alberto Gallo, Marco Spezia, Antonio Pompilio Gigante

**Affiliations:** 1 Present Address: Clinical Orthopedics, Department of Clinical and Molecular Science, School of Medicine, Università Politecnica delle Marche, Via Tronto, 10/a, 60126 Ancona, AN Italy; 2 Present Address: Orthopedic Unit, Habilita Casa di Cura I Cedri, Via Don Guanella, 1, 28073 Fara Novarese, NO Italy

**Keywords:** Creatine kinase, C-reactive protein, Direct anterior approach, Posterolateral approach, Minimally-invasive surgery, Total hip arthroplasty

## Abstract

**Background:**

This retrospective study compares the invasiveness of the direct anterior approach (DAA) and the posterolateral approach (PLA) in total hip arthroplasty (THA) by assessing three widely used inflammation-related serum markers in the first ten post-operative days.

**Methods:**

The database of our institution was mined for primary THAs conducted by the DAA or the PLA from February 2020 to June 2022. Demographics and creatine kinase (CK), C-reactive protein (CRP), and white blood cells were compared. Propensity Score Matching (PSM) analysis (1:1 ratio) was conducted based on multiple variables.

**Results:**

PSM analysis yielded 44 pairs of DAA and PLA patients. CK was significantly higher (*p* < 0.001) in the DAA than in the PLA group on postoperative day (POD) 2, 5 and 10. The POD2, POD5 and POD10 CK/preoperative CK ratio was 12.9, 5.0 and 0.8 in DAA and 8.8, 3.3 and 0.6 in PLA (*p* = 0.017, *p* = 0.012 and *p* = 0.025, respectively). The POD2, POD5 and POD10 CRP/preoperative CRP ratio was 95.1, 65.6 and 22.8 in PLA and 34.7, 23.3 and 8.9 in DAA (*p* < 0.001, *p* = 0.002 and *p* < 0.001, respectively).

**Conclusion:**

PSM analysis of early postoperative CK and CRP values demonstrated that the DAA should be considered as a less stressful approach, not as a muscle-sparing or a minimally invasive THA approach.

## Background

Total hip arthroplasty (THA), one of the most effective orthopedic procedures devised in recent decades, is the treatment of choice for symptomatic end-stage osteoarthritis [1]. Over time, clinical outcomes have continuously improved and implant survival rates have increased to over 95% at ten years [2, 3]. As population aging and the spread of obesity boost the requirements for THA procedures, there is as yet no agreement on the best surgical approach to the hip joint for primary THA, since all methods have advantages as well as drawbacks [4]. In recent years, “minimally invasive” approaches, which offer faster recovery and less postoperative pain, though involving a learning curve, have become increasingly popular [5–10]. In particular, the direct anterior approach (DAA), where joint exposure is performed through internervous and intermuscular planes [11], has been reported to provide better early postoperative outcomes [12–14], although the literature is inconclusive.

C-reactive protein (CRP) is currently employed as a useful measure of inflammation and infection, being considered as a more reliable marker of infection-related surgical complications than erythrocyte sedimentation rate and white blood cell (WBC) count [15–17]. The CRP response objectifies the surgical trauma and can thus be used to quantify its invasiveness, assess tissue damage, and monitor perioperative stress [18]. THA and total knee arthroplasty induce leukocytosis. In THA, total leukocyte counts have been reported to peak on postoperative day (POD)1, whereas the WBC count appeared to decline on POD5, but did not revert to preoperative values [19, 20].

Serum enzymes have recently been proposed as objective measures of the muscle damage and inflammation induced by surgical procedures [21–24]. Indeed, elevation of serum creatine kinase (CK) after orthopedic surgery has been described even in the absence of myocardial damage [25, 26]. The claim that the DAA to the hip joint, which avoids muscle resection by passing through intermuscular and internervous planes, is a minimally invasive approach has been substantiated by the lower CK levels found in DAA patients compared to individuals subjected to posterior approaches. However, conflicting findings were previously reported. Additionally, large heterogeneity was found in the comparison groups making the findings hard to generalize.

Therefore, this study aimed to the first study that uses Propensity Score Matching analysis to examine whether the DAA is less invasive than the PLA by comparing three widely used serum markers of inflammation over 10-day follow-up.

## Methods

### Patient selection

The records of the patients who underwent primary THA by the DAA or the PLA from February 2020 to June 2022 and for whom a follow-up of at least 10 days was available were retrospectively retrieved from the institutional database. The data collected included demographics, body mass index (BMI), medical history, current treatments, preoperative diagnosis, inpatient history, American Society of Anesthesiologists (ASA) class, type of anesthesia, operative time, pre- and postoperative serum CK, CRP, and WBC values, any intraoperative complications, and any infections up to 3 months after surgery.

### Inclusion and exclusion criteria:

Participants were patients aged 18–70 years who underwent THA (by the DAA or the PLA) for primary degenerative hip osteoarthritis. Exclusion criteria were unwillingness to participate; a BMI ≥ 35; inflammatory arthropathy, autoimmune or rheumatic disease; previous procedures involving the affected hip; bilateral hip arthroplasty; contralateral THA and any arthroplasty procedure; a diagnosis of congenital/acquired muscle disease, ischemic cardiac disease, or end-stage renal failure; hepatitis, liver disease, or malignancy; a history of cerebrovascular disease; peripheral neuropathy; cognitive deficits; a recent history of rhabdomyolysis; current treatment with immunosuppressive or myotoxic drugs; urinary tract infection; contact with COVID-19 patients during hospitalization; internal or surgical intraoperative complications requiring cement or cerclage; postoperative wound or upper/lower respiratory tract infection; implant infection up to 3 months after THA; postoperative complications such as venous thrombosis, hematoma or fever (> 38 °C). Patients whose serum samples had been analyzed elsewhere were excluded. Patients older than 70 years were also excluded, due to their atypical response to surgical stress [27] moreover, since these subjects might already suffer from muscle damage, they are hypersensitive to even minor muscle injury [28, 29]. All patients provided their signed informed consent.

### Indications for surgery

All patients had been diagnosed with hip osteoarthritis based on history, physical examination, and imaging findings. If after three months of conservative treatment (physical therapy, intra-articular cortisone injections, rest, and anti-inflammatory drugs) they still complained of significant pain, they were offered hip arthroplasty by the DAA or the PLA and given the same preoperative education program.

### Surgical procedure

THA was performed by two senior orthopedic surgeons (MS for DAA, AG for PLA), whose extended experience in the respective approach (> 15 years) far exceeds the learning curve [30, 31]. All patients received dual-mobility implants with an uncemented acetabular component (Acorn Primary Dual Mobility), an uncemented femoral component (Exacta short, lateralized or standard femoral stem), a 28 mm metal femoral head (ceramic in patients with metal allergy or sensitivity), and a polyethylene insert (ACORN dual mobility cup insert), all from Permedica, Merate, Italy.

### Perioperative procedures

All patients received perioperative antibiotic prophylaxis (cefazolin 2 g) and 1 g intravenous tranexamic acid 20 min before the skin incision and anesthesia (general or spinal). Postoperative pain control included intravenous tramadol 20 mg + metoclopramide 10 mg for 24 h. Over the next few days oral paracetamol or oral tramadol were administered as needed. Postoperative thromboembolic prophylaxis was ensured by low molecular weight heparin and use of elastic stockings (both limbs). Physiotherapy was begun on POD1. Weightbearing and walking with aids were allowed the next morning, after an x-ray check of implant positioning and bone integrity. In POD 2, all patients were transferred to the hospital's rehabilitation department where they continued their inpatient physical therapy course.

### Serum levels

The aim of the study was to compare the serum values of three widely used inflammatory markers before and after the procedure and between the DAA and the PLA. CK and CRP were determined before the operation and on PODs 2, 5 and 10, whereas WBC was evaluated preoperatively and on PODs 1, 2, 5, and 10 [32, 33]. The ratio of postoperative to preoperative levels of the three markers was calculated and compared in the two groups.

### Statistical analysis

All analysis were conducted using Microsoft Excel (Microsoft) with the XLSTAT resource pack (XLSTAT-Premium, Addinsoft, New York, USA). A Propensity Score Matching (PSM) analysis was conducted to adjust for the differences in known covariates between the groups [34]. Patients treated by the DAA and the PLA were matched 1:1 using an optimal matching algorithm [35] which determines the matched samples with the smallest average absolute distance across all matched pairs. This type of matching, which is considered as an ideal method to assess differences between treatment groups was used to reduce the effect of potential confounding variables between DAA and PLA individuals [36]. Patients were eligible for matching if the difference of the propensity score between DAA and PLA was within the caliper radius of 0.01 × sigma. The strength of the association was estimated with 95% confidence intervals. The variables on which the two groups were matched included gender, type of anesthesia, and use of drainage (categorical data) and age, BMI, operative time and preoperative levels of CK, CRP and WBC (quantitative data). The Shapiro–Wilk test was performed to assess whether the data showed a normal distribution. Calculated mean values and SMD (standardized mean difference) were also reported for all continuous data. A non-parametric test (Mann–Whitney for unpaired data and Wilcoxon signed rank for paired data) was applied to assess continuous variables for significant differences between the groups. The categorical data were subjected to the chi-square test. Imbalances between the DAA and PLA groups were identified by comparing the SMD before and after the matching. A group was considered imbalanced for a particular covariate if SMD was > 0.2 [34].

A *p* value < 0.05 was considered significant. The statistical analyses were conducted using Microsoft Excel (Microsoft) and the XLSTAT resource pack.

## Results

**Fig. 1:**
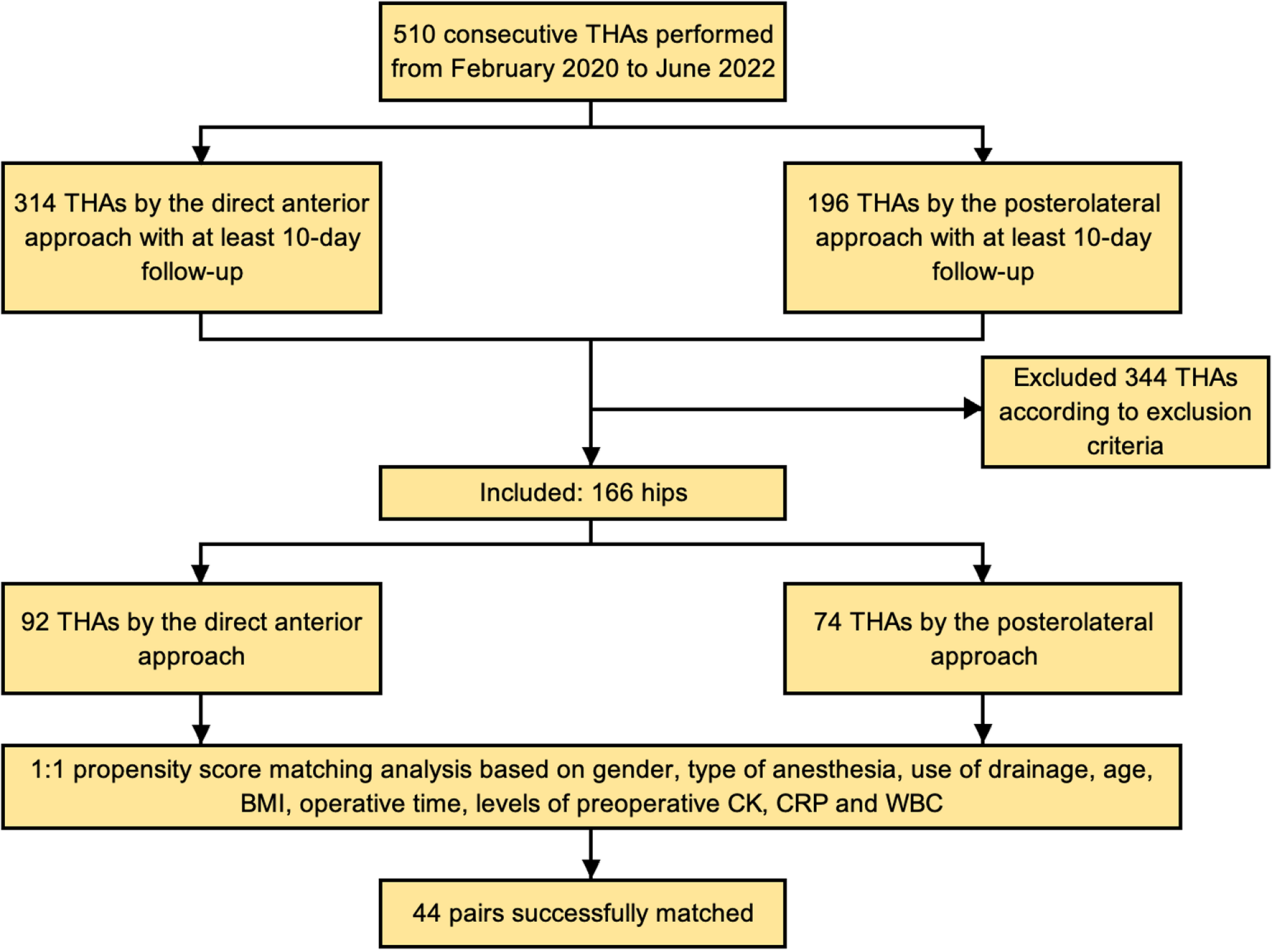
Patient selection flowchart. THA: total hip arthroplasty

According to the institutional database, 510 THAs with 10-day follow-up, 314 conducted by the DAA and 196 by the PLA, were performed at our institution from February 2020 to June 2022. A total number of 92 DDA patients and 74 PLA patients met the inclusion/exclusion criteria. PSM analysis yielded 44 pairs of patients who were successfully matched for gender, type of anesthesia, drainage use, age, BMI, operative time and preoperative levels of CK, CRP and WBC (Fig. 1).

### Patient data

Before PSM analysis, the two groups (92 DAA and 74 PLA patients) were imbalanced regarding BMI and operative time with SMD values of 0.82 and 0.45 respectively. The two groups had significantly different BMI (*p* < 0.001), ASA class (*p* < 0.001), and operative time (*p* < 0.001). PSM analysis, where patients were matched 1:1, yielded two similar groups that did not have significantly different preoperative, perioperative or postoperative features (Table 1).

**Table 1:** Preoperative, perioperative, and postoperative data of the THA patients

Variable	Before propensity score matching				After propensity score matching			
	DAA	PLA	p value	SMD	DAA	PLA	p value	SMD
Age, mean (SD) [range]	62.9 (7.3) [46–70]	63.0 (6.1) [50–70]	0.441	0.01	61.8 (7.7) [46.00–70.00]	63.2 (5.5) [52–69]	0.317	0.20
Gender								
Male (%)	54 (58.7)	42 (56.8)	0.801	0.04	28 (63.6)	24 (54.5)	0.386	0.18
Female (%)	38 (41.3)	32 (43.2)			16 (36.4)	20 (45.5)		
Side:								
Right (%)	42 (45.7)	34 (46.0)	0.97		19 (43.2)	22 (50.0)	0.521	
Left (%)	50 (54.3)	40 (54.0)			25 (56.8)	22 (50.0)		
BMI (kg/m^2^), mean (SD) [range]	25.2 (3.1) [17–30]	27.3 (1.9) [22.5–29.8]	< 0.001	0.82	27.3 (1.9) [23.2–30.0]	26.7 (2.0) [22.5–29.8]	0.201	0.30
Smoking status:								
Never (%)	72 (78.3)	56 (75.7)	0.694		34 (77.3)	30 (68.2)	0.338	
Current smoker (%)	20 (21.7)	18 (24.3)			10 (22.7)	14 (31.8)		
Alcohol:								
Never (%)	24 (26.1)	16 (21.6)	0.504		8 (18.2)	8 (18.2)	1.000	
With meals (%)	68 (73.9)	58 (78.4)			36 (81.8)	36 (81.8)		
ASA class (%)								
ASA 1	30 (32.6)	26 (35.1)			10 (22.7)	16 (36.4)		
ASA 2	54 (58.7)	26 (35.1)	< 0.001		26 (59.1)	16 (36.4)	0.102	
ASA 3	8 (8.7)	22 (29.8)			8 (18.2)	12 (27.2)		
Anesthesia								
General (%)	4 (4.4)	4 (5.4)	0.752	0.01	2 (4.5)	2 (4.5)	1.000	0
Spinal (%)	88 (95.7)	70 (94.6)			42 (95.5)	42 (95.5)		
Operative time (min), mean (SD) [range]	57.3 (13.1) [40–90]	62.7 (10.5) [45–80]	< 0.001	0.45	62.2 (12.7) [40.0–90.0]	63.4 (10.6) [45–80]	0.379	0.10
Drain								
Yes (%)	78 (84.8)	60 (81.1)	0.527	0.10	34 (77.3)	30 (68.2)	0.338	0.20
No (%)	14 (15.2)	14 (18.9)			10 (22.7)	14 (31.8)		
Length of hospital stay (days), mean (SD) [range]	12.8 (1.9) [10–18]	13.2 (2.1) [10–18]	0.327		12.7 (1.7) [10–16]	12.6 (2.0) [10–16]	0.857	

*THA* total hip arthroscopy; *DAA* direct anterior approach; *PLA* posterolateral approach; *SD* standard deviation; *BMI* body mass index; *ASA* American Society of Anesthesiology; *SMD* standardized mean difference

After PSM analysis, a residual imbalance remained regarding BMI (SMD = 0.30), but there were no significant differences (*p* = 0.201).

### Serum marker values

Before matching, the DAA and PLA group were imbalanced regarding preoperative CRP values (SMD = 0.30); after matching a residual imbalance remained (SMD = 0.28).

The DAA and PLA groups had not significantly different levels of preoperative CK, CRP, or WBC either before or after matching.

As regards the postoperative values, serum CK on POD2 was 1619.5 ± 936.0 IU/l (range, 344.0–3902.0) in the DAA group and 740.9 ± 401.7 IU/l (range, 124.0–1756.0) in the PLA group (p < 0.001). Serum CK on POD5 was 690.5 ± 489.0 IU/l (range, 98.0–2453.0) in the DAA group and 326.4 ± 260.9 IU/l (range, 66.0–993.0) in the PLA group (*p* < 0.001). Serum CK on POD10 was 103.3 ± 65.6 IU/l (range, 44.0–353.0) in the DAA group and 74.3 ± 55.3 IU/l (range 22.0–234.0) in the PLA group (*p* < 0.001). The ratio of POD2 CK to preoperative CK was 12.9 ± 8.2 (range, 3.4–44.3) in the DAA group and 8.8 ± 8.3 (range, 1.1–36.6) in the PLA group (p = 0.017). The ratio of POD5 CK to preoperative CK was 5.0 ± 3.4 (range, 1.8–16.4) in the DAA group and 3.3 ± 3.2 (range, 0.4–16.8) in the PLA group (*p* = 0.012). The ratio of POD10 CK to preoperative CK was 0.8 ± 0.5 (range, 0.2–1.9) in the DAA group and 0.6 ± 0.2 (range, 0.2–1.0) in the PLA group (*p* = 0.025).

Serum CRP on POD2 was 3.7 ± 0.6 mg/dl (range, 2.1–6.5) in the DAA group and 8.6 ± 1.5 mg/dl (range, 6.1–11.6) in the PLA group (*p* < 0.001). Serum CRP on POD5 was 2.6 ± 1.0 mg/dl (range, 0.7–4.3) in the DAA group and 5.7 ± 1.2 mg/dl (range, 4.0–8.1) in the PLA group (*p* < 0.001), whereas on POD10 it was 1.0 ± 0.6 mg/dl (range, 0.3–2.3) in the DAA group and 2.5 ± 1.0 mg/dl (range, 0.8–4.8) in the PLA group (*p* < 0.001). The POD2 CRP/preoperative CRP ratio was 34.7 ± 24.1 (range 4.7–114.0) in the DAA group and 95.1 ± 174.5 (range, 13.8–1116.0) in the PLA group (*p* < 0.001). The POD5 CRP/preoperative CRP ratio was 23.3 ± 19.1 (range 3.9–95.7) in the DAA group and 65.6 ± 124.5 (range, 8.0–764.0) in the PLA group (*p* = 0.002). The POD10 CRP/preoperative CRP ratio was 8.9 ± 7.2 (range, 1.5–30.5) in the DAA group and 22.8 ± 25.2 (range 1.6–135.0) in the PLA group (*p* < 0.001).

No significant differences in WBC values and ratios were found in serum except for POD1 and POD5 WBC/preoperative WBC ratio (*p* = 0.041 and *p* = 0.022, respectively) (Table 2).

**Table 2:** Comparison between DAA and PLA group

Variable	Before propensity score matching				After propensity score matching			
	DAA	PLA	p value	SMD	DAA	PLA	p value	SMD
*CK (IU/l)*								
Preoperative, mean (SD) [range]	141.5 (99.7) [22–440]	134.4 (98.2) [28.0–389.0]	0.379	0.07	149.3 (90.95) [44.0–413.0]	131.9 (94.4) [28.0–371.0]	0.276	0.18
POD2, mean (SD) [range]	1492.2 (1032.0) [121.0–5208.0]	736.9 (431.2) [124.0–2396.0]	< 0.001		1619.5 (936.0) [344.0–3902.0]	740.9 (401.7) [124.0–1756.0]	< 0.001	
POD5, mean (SD) [range]	769.2 (657.8) [98.0–3430.0]	335.7 (280.5) [41.0–1258.0]	< 0.001		690.5 (489.0) [98.0–2453.0]	326.4 (260.9) [66.0–993.0]	< 0.001	
POD10, mean (SD) [range]	85.1 (58.1) [19.0–353.0]	71.0 (46.9) [22.0–234.0]	0.029		103.3 (65.6) [44.0–353.0]	74.3 (55.3) [22.0–234.0]	< 0.001	
Ratio of POD2 CK/Preop CK, mean (SD) [range]	13.8 (14.4) [2.2–98.3]	8.3 (7.5) [1.0–36.6]	< 0.001		12.9 (8.2) [3.4–44.3]	8.8 (8.3) [1.1–36.6]	0.017	
Ratio of POD5 CK/Preop CK, mean (SD) [range]	6.7 (7.1) [1.0–44.0]	3.2 (3.0) [0.4–16.8]	< 0.001		5.0 (3.4) [1.8–16.4]	3.3 (3.2) [0.4–16.8]	0.012	
Ratio of POD10 CK/Preop CK, mean (SD) [range]	0.7 (0.4) [0.2–1.9]	0.6 (0.2) [0.2–1.0]	0.441		0.8 (0.5) [0.2–1.9]	0.6 (0.2) [0.2–1.0]	0.025	
*CRP (mg/dl)*								
Preoperative, mean (SD) [range]	0.2 (0.1) [0.02–0.70]	0.2 (0.2) [0.01–0.6]	0.032	0.30	0.18 (0.14) [0.03–0.7]	0.21 (0.16) [0.01–0.6]	0.215	0.28
POD2, mean (SD) [range]	3.6 (0.9) [1.4–6.5]	8.5 (1.6) [6.1–12.5]	< 0.001		3.7 (0.6) [2.1–6.5]	8.6 (1.5) [6.1–11.6]	< 0.001	
POD5, mean (SD) [range]	2.7 (1.1) [0.7–5.8]	5.5 (1.3) [3.3–8.3]	< 0.001		2.6 (1.0) [0.7–4.3]	5.7 (1.2) [4.0–8.1]	< 0.001	
POD10, mean (SD) [range]	1.0 (0.6) [0.2–2.3]	2.2 (1.0) [0.6–4.8]	< 0.001		1.0 (0.6) [0.3–2.3]	2.5 (1.0) [0.8–4.8]	< 0.001	
Ratio of POD2 CRP/Preop CRP, mean (SD) [range]	37.8 (26.0) [4.6–114.7]	89.7 (141.0) [13.8–1116.0]	< 0.001		34.7 (24.1) [4.7–114.0]	95.1 (174.5) [13.8–1116.0]	< 0.001	
Ratio of POD5 CRP/Preop CRP, mean (SD) [range]	27.7 (20.6) [3.8–96.3]	58.6 (99.4) [7.6–764.0]	< 0.001		23.3 (19.1) [3.9–95.7]	65.6 (124.5) [8.0–764.0]	0.002	
Ratio of POD10 CRP/Preop CRP, mean (SD) [range]	10.3 (8.6) [1.5–40.0]	19.5 (21.1) [1.6–135.0]	< 0.001		8.9 (7.2) [1.5–30.5]	22.8 (25.2) [1.6–135.0]	< 0.001	
*WBC (× 10^9 l)*								
Preoperative, mean (SD) [range]	7.1 (1.8) [4.5–13.5]	6.9 (1.7) [3.8–10.4]	0.96	0.11	7.0 (1.8) [4.6–13.5]	6.9 (1.7) [3.8–10.0]	0.968	0.06
POD1, mean (SD) [range]	9.3 (2.5) [4.7–19.7]	10.7 (3.5) [6.2–23.2]	0.008		9.3 (2.4) [5.6–19.7]	10.0 (2.3) [6.2–15.3]	0.096	
POD2, mean (SD) [range]	8.6 (1.9) [4.6–12.7]	9.9 (2.9) [5.9–19.7]	0.011		8.8 (1.6) [4.9–11.1]	9.1 (1.8) [5.9–12.9]	0.610	
POD5, mean (SD) [range]	6.3 (1.2) [3.9–8.6]	7.2 (1.9) [4.0–13.9]	0.001		6.4 (1.0) [4.6–8.6]	6.9 (1.5) [4.0–10.4]	0.096	
POD10, mean (SD) [range]	7.0 (1.1) [4.4–9.7]	7.6 (2.5) [4.3–18.3]	0.077		6.8 (0.9) [4.4–9.6]	7.2 (1.6) [4.3–9.7]	0.201	
Ratio of POD1 WBC/Preop WBC, mean (SD) [range]	1.3 (0.2) [1.0–2.0]	1.6 (0.4) [1.0–2.3]	< 0.001		1.4 (0.3) [1.0–2.0]	1.5 (0.4) [1.0–2.2]	0.041	
Ratio of POD2 WBC/Preop WBC, mean (SD) [range]	1.3 (0.3) [0.7–2.0]	1.5 (0.4) [0.9–2.3]	< 0.001		1.3 (0.2) 0.8–1.6]	1.4 (0.4) [0.9–2.2]	0.215	
Ratio of POD5 WBC/Preop WBC, mean (SD) [range]	0.9 (0.2) [0.6–1.2]	1.1 (0.2) [0.7–1.6]	< 0.001		0.9 (0.1) [0.6–1.2]	1.0 (0.2) [0.7–1.4]	0.022	
Ratio of POD10 WBC/Preop WBC, mean (SD) [range]	1.0 (0.2) [0.6–1.7]	1.1 (0.3) [0.6–2.2]	0.024		1.0 (0.2) [0.6–1.3]	1.1 (0.3) [0.6–1.6]	0.147	

*DAA* direct anterior approach; *PLA* posterolateral approach; *CK* creatine kinase; *POD* postoperative day; *CRP* C-reactive protein; *WBC* white blood cells; *SMD* standardized mean difference

## Discussion

This study set out to compare the invasiveness of THA, conducted by the DAA or the PLA, by analyzing three widely used serum markers of inflammation and muscle injury in a sample of DAA and PLA patients matched by PSM analysis. Our chief finding was that the DAA involved significantly higher CK and significantly lower CRP values than the PLA. In particular, serum CK was significantly higher on POD2, POD5 and POD10 in the DAA group, where the ratio of POD 2 and POD5 CK to preoperative CK was higher that the PLA group. As regards CRP, on POD2, POD5 and POD10 it was significantly higher in the PLA group, where the POD2 and POD5 CRP/preoperative CRP ratio was about more than 3 times greater, while the POD10 CRP/preoperative CRP ratio was almost twice compared to that of the DAA group. In contrast, the WBC counts never showed significant differences, suggesting that this measure may not be critical for the assessment of procedure invasiveness. Data analysis highlighted that the two patient groups showed no significant differences in serum CK or CRP both before and after matching. Far from being a drawback or a bias, this finding demonstrates that before THA the two groups were in fact very similar.

In recent years, “minimally invasive” THA approaches have gained considerable popularity for their ability to ensure a swifter recovery and a less painful postoperative course, despite a not negligible risk of complications. In particular, the DAA has been reported to provide better clinical outcomes, reduced painkiller use, and shorter hospital stays [5], although it is burdened by a long learning curve and a higher risk of lateral femoral cutaneous nerve injury and iatrogenic fractures compared with other approaches [37].

CRP has been proposed as a measure of the overall invasiveness of surgical procedures, particularly of tissue damage and perioperative stress [18, 33]. Our DAA patients had significantly lower postoperative CRP than the PLA group both before and after matching; this contrasts with several studies describing comparable CRP values in patients managed by the two approaches [38, 39]. As regards CK, reports of its value as a measure of invasiveness are inconsistent [23, 24, 38, 40–43]. In particular, one study has demonstrated that patients managed by the PLA had higher CK levels in the early postoperative days than patients managed by the DAA [41], whereas another found that in the immediate postoperative period CK values were 5.5 times higher in PLA patients [24]. In a prospective randomized study of patients subjected to DAA or PLA, [38] CK and CRP did not show significant differences between the groups either before surgery and at 6 weeks. The CK values found in our patients are in line with those described by Maezawa et al. [39], who measured CK and CRP preoperatively, immediately after surgery, and then on PODs 1 and 4 in patients managed by the DAA or the PLA. This finding may be explained by the stress induced by the retraction of the tensor fasciae latae, rectus femoris, sartorius, and gluteus medius, despite the fact that the DAA does not involve muscle resection. Furthermore, any injury to these muscles during broaching of the proximal femur or stem implantation results in serum marker elevation [40, 41, 44, 45].

For these reasons, the DAA has a greater potential for muscle damage than other approaches [40], as demonstrated by Meneghini et al. [46] for the rectus femoris and the tensor fasciae latae and by Van Oldenrijk et al. [47] for the lateral femoral cutaneous nerve. In contrast, Frye et al. [48] found that muscle injury in the DAA is more frequent in men patients with a high BMI. As regards the WBC count, values were not significantly different in the two groups. However, the change after THA was in line with the one reported by Hughes et al. [19] and Høgevold et al. [20].

In our opinion THA through DAA should not be considered as a muscle-sparing or minimally invasive approach, but mainly as a less stressful approach for the patient. Indeed, CRP, which assesses the systemic response to surgical stress, was consistently lower, while CK, marker of locally produced muscle damage, was significantly higher. These data may suggest proposing THA through DAA in patients with increased surgical risk, as it has less impact on the systemic response to surgery.

To the best of our knowledge, this is the first work where the PSM strategy is applied to compare three widely used serum markers in DAA and PLA patients subjected to primary THA. The main strength of our study is that the procedures were performed by two high-volume surgeons with strong experience in their chosen approach [49, 50] on patients who followed the same preoperative protocol, received the same implant, and were managed by the same rehabilitation protocol. Other significant advantages are the application of exacting inclusion and exclusion criteria, which ensured a very homogeneous patient sample, and the use of PSM, which further reduced confounding biases, although it clearly yielded a small number of patient pairs.

The chief limitation of our study is its retrospective and non-randomized design. Pair matches obtained were only 44, and the study may be underpowered. However, considering an effect size of 0.3, and α level with *p* = 0.05, the Post hoc power analysis was 86% (G-Power version 3.1, Düsseldorf, Germany) [51].

That fact that we did not examine patients’ clinical outcomes may also be considered as a limitation; however, since the only purpose of the study was to compare the invasiveness of the two THA approaches, the reader is referred to the literature for the functional outcomes [52]. Moreover, the length of skin incision was not considered in the statistical analysis. Finally, the decision to analyze CK, CRP, and WBC, but not other serum markers such as interleukins, myoglobin, and tumor necrosis factor alpha [23, 24, 32, 41, 53], stems from the inconclusive data on their value.

Age plays a well-documented role in serum enzyme levels [27]. To minimize its influence, we excluded patients aged more than 70 years, who show atypical responses to surgical stress [27], and applied the PSM strategy, which further reduced confounding biases. Since biochemical markers can be affected a variety of factors, further approaches capable of quantifying tissue damage should be developed and honed [54–56]. For instance, diagnostic imaging methods such as MRI can supply additional useful information on the invasiveness of surgical procedures in general and of THA approaches in particular.

## Conclusions

In conclusion, the main finding of this study was that two widely used serum markers of inflammation and muscle injury increase in the first postoperative days both in THA patients managed by the DAA and in those managed by the PLA. In the DAA group, CRP was consistently lower, whereas CK was significantly higher on POD5 and then reverted to baseline within 10 days. For these reasons, the DAA should be considered as a less stressful rather than as a minimally invasive THA approach.

## Data Availability

The datasets used and/or analysed during the current study are available from the corresponding author, LDB, on reasonable request.

## Abbreviations

DAA: Direct anterior approach
THA: Total hip arthroplasty
PLA: Posterolateral approach
CK: Creatine kinase
CRP: C-reactive protein
WBC: White blood cells
PSM: Propensity score matching
POD: Postoperative day
